# Urgent Need for Tobacco Cessation Initiatives Among LGBT Communities in Nepal

**Published:** 2024-12-14

**Authors:** Kiran Paudel, Aashish Rana, Kamal Gautam, Aney Rijal, Bhakta KC, Tara Ballav Adhikari, Roman Shrestha

**Affiliations:** 1 Nepal Health Frontiers, Tokha-5, Kathmandu, Nepal; 2 Department of Allied Health Sciences, University of Connecticut, Storrs, CT 06269, USA; 3 Central Department of Public Health, Institute of Medicine, Tribhuvan University, Kathmandu, Nepal; 4 HERD International, Lalitpur, Nepal; 5 Ministry of Health and Population, Government of Nepal; 6 Research Unit for Global Health, Department of Public Health, Aarhus University, Aarhus, Denmark

## Introduction

The World Health Organization (WHO) estimated that approximately 22.3% of the world’s population used tobacco products in 2020, killing more than 8 million people annually, which remains a global public health challenge [1]. This issue is alarming in low-middle-income countries (LMIC), where approximately 80% of the 1.3 billion tobacco users worldwide reside in low- and middle-income countries, accounting for 78% of tobacco-related deaths [1–3]. The Non-communicable Disease Risk Factors: STEPS Survey Nepal 2019 revealed that more than one-fourth (28.9%) of adults aged 15 to 69 years consumed tobacco products [4].

Globally, smoking prevalence is disproportionately high among LGBT youth (38-59%) compared to the general youth population (28-35%) [5]. Smoking is also more common in this population because it is socially accepted and many start smoking at younger ages compared to the general population [5, 6]. There are few LGBT-friendly social spaces, such as bars and clubs, which play a vital role in fostering community connections. However, these venues often have higher smoking rates [7]. In addition, tobacco companies have heavily targeted this group through ads and sponsorship using themes like rainbow-colored e-cigarettes and supporting pride events. Furthermore, LGBT individuals often face higher stress due to discrimination, stigma, and the challenge of coming out which can lead to smoking as a coping mechanism [6, 8].

Findings from the Global Burden of Disease 2021 report highlighted that smoking is ranked as the second leading preventable risk factor for disease [9]. Cigarette smoke contains more than 7,000 chemicals, of which at least 69 cause cancer [10]. Cigarette smoking causes cancer, heart disease, stroke, lung diseases such as chronic obstructive pulmonary disease (COPD), type 2 diabetes, reproductive health problems and other health problems [11]. For the LGBT community, these risks are compounded by the existing health disparities [12]. LGBT are at higher risk for health issues including HIV/AIDs, sexually transmitted infections, and mental health challenges which might take precedence over concerns related to tobacco use [12]. Many LGBT individuals in Nepal face barriers in accessing healthcare due to fear of discrimination, lack of financial resources, or inadequate knowledge about available services [13–15]. This results in the delayed diagnosis and treatment of smoking-related illnesses and smoking cessation.

### Smoking cessation among LGBT

Approximately two-thirds of the LGBT intend to quit smoking which was higher than the general population [16]. However, there are no documented shreds of evidence on LGBT related to smoking cessation in Nepal. There might be several barriers that hinder smoking cessation efforts in Nepal’s LGBT community. First, cultural stigma and discrimination discourage many individuals from seeking help, as they fear judgment or mistreatment in healthcare settings [14, 15]. Second, existing smoking cessation programs such as the role of minority stress in tobacco use are not designed to address the unique needs of LGBT individuals. Third, limited awareness of the risks of smoking and the availability of cessation resources further complicates this issue. In addition, withdrawal symptoms, end-of-month disputes, and LGBT individuals are more frequently exposed to secondhand smoke compared to non-LGBT counterparts which reduces the likelihood of smoking cessation.

While Nepal has implemented several tobacco control initiatives, excelling in several components of the WHO’s EMPOWER measures, it lags significantly in cessation efforts [17]. The limited availability of cessation programs (Nicotine Replacement Therapy), currently available in two hospitals in the country’s capital, suggests a gap in accessible treatment for tobacco dependence [18]. One of the important therapeutic interventions of smoking cessation services (bupropion, and varenicline) is provided exclusively by psychiatrists [19]. However, with only 200 psychiatrists nationwide (0.68 psychiatrists per 100,000), the country faces a significant shortage of trained clinicians [20]. The available programs have been insufficiently tailored to meet the unique needs of LGBT, and therefore, they are less likely to seek out such services. LGBT who seek to quit smoking and visit LGBT-friendly community-based health clinics often find these clinics lack such services. Such clinics are critical settings for smoking cessation among LGBT because they are often the first and only point of contact with the healthcare system. Therefore, there is an urgent need to improve access to evidence-based and highly scalable smoking cessation interventions, specifically tailored to address the unique needs of LGBT.

### Recommendations

Addressing smoking in Nepal’s LGBT community requires a multifaceted approach that combines research, policy advocacy, and community engagement. Below are some key recommendations:

1. Conduct Research: National-level research initiatives such as the Nepal Demographic and Health Survey and the Non-communicable Disease Risk Factors: STEPS Survey Nepal do not address the prevalence and patterns of smoking and tobacco use within the LGBT community. There is an urgent need for Nepal-specific studies to explore smoking behaviors, underlying factors, and cessation requirements in the LGBT population. Such data are crucial and a benchmark for designing tailored interventions to address these issues effectively.
2. Raise Awareness: Public health initiatives should focus on informing the LGBT community about the advantages of quitting. These efforts should also emphasize the resources and support networks that are accessible to them. Some of the examples are: i) Distribution of multilingual brochures/videos in clinics, pride events, and online platforms highlighting cessation benefits and support services (e.g., quitlines, mental health hotlines). ii) Utilization of social media (e.g., Facebook, TikTok, YouTube) and mass media (e.g., radio, television, newspapers) to share stories of sexual & gender minority iindividuals who quit smoking, collaborating with influencers.
3. Engage the Community: LGBT organizations and leaders should be actively involved in designing and implementing cessation programs. The tobacco cessation programs should be channeled through their organization, by empowering them to make effective implementation of the tobacco cessation programs. Community-based approaches are more likely to be effective and sustainable.
4. Develop Tailored Cessation Programs: Smoking cessation programs must be culturally sensitive and inclusive to address the unique challenges faced by LGBT individuals. Brief tobacco intervention for LGBT smokers is one of the cost-effective interventions for smoking cessation. Similarly, hotline service and mHealth have enormous potential for reaching and supporting individuals at heightened risk of smoking relapse as smartphone use is widespread among MSM (97.9%), in Nepal. However, such mHealth interventions are predominantly designed for general populations in high-income countries, limiting their relevance in LMIC contexts and their ability to address the unique needs of LGBT individuals.
5. Advocate for tobacco Policy implementation: The reforms mandated by the Tobacco Products Control and Regulatory Act 2011 must be effectively translated into action [21]. Additionally, the reforms should include non-discriminatory service provisions, such as implementing awareness programs, utilizing healthcare tax as prescribed, and integrating LGBT cultural competency into medical curricula.

Additionally, amendments should be made to mandate equal access to healthcare services and allocate funding for LGBT-specific cessation programs. Policymakers must prioritize tobacco control measures and work toward making healthcare services more accessible and inclusive for marginalized communities. This requires training healthcare providers to deliver nonjudgmental, culturally competent care.

## Conclusion

The high prevalence of smoking, coupled with existing health disparities, underscores the need for targeted interventions that address the root causes of tobacco use. There is a critical gap in research on smoking and cessation among LGBT populations, which is essential for informing interventions. Addressing this gap could provide a foundation for stakeholders to develop targeted policies and programs. By fostering collaboration between the government, NGOs, healthcare providers, and the LGBT community, Nepal can take meaningful steps toward reducing smoking rates and improving the overall health and well-being of its LGBT population. Among them, smoking cessation is a critical public health intervention that needs to be implemented/provided immediately in Nepal’s LGBT community.
